# Backward Aortic Flow Is Associated with Impaired Left Ventricular Mechanics in Young Adults with Bicuspid Aortic Valve: A Prospective Cardiovascular Magnetic Resonance Study

**DOI:** 10.3390/jcm15176828

**Published:** 2026-09-03

**Authors:** Oana Iulia Man, Rares Ilie Orzan, Dalma Horvat, Renata Agoston, Andra Negru, Cecilia Lazea, Silvia Lupu, Lucia Agoston-Coldea

**Affiliations:** 1 Department of Internal Medicine, Iuliu Hatieganu University of Medicine and Pharmacy, 400012 Cluj-Napoca, Romania; 2 Department of Pediatrics, Iuliu Hatieganu University of Medicine and Pharmacy, 400347 Cluj-Napoca, Romania; 3 Internal Medicine V, George Emil Palade University of Medicine, Pharmacy, Science, and Technology of Targu Mures, 540139 Targu Mures, Romania; 4 1st Department of Cardiology, Emergency Institute for Cardiovascular Disease and Heart Transplant of Targu Mures, 540136 Targu Mures, Romania; 5 Department of Radiology, Affidea Hiperdia Diagnostic Imaging Center, 400012 Cluj-Napoca, Romania; 6 2nd Department of Internal Medicine, Emergency County Hospital, 400012 Cluj-Napoca, Romania

**Keywords:** cardiovascular magnetic resonance, bicuspid aortic valve, left ventricular global longitudinal strain, left ventricular torsion

## Abstract

**Objectives:** The presence of a bicuspid aortic valve (BAV) is known to alter transvalvular flow and increase shear stress on the ascending aorta, contributing to alterations in aortic geometry and the progression of BAV-related aortopathy. The purpose of the current study was to characterize aortic flow abnormalities and their associations with aortic geometry and ventricular mechanics using cardiac magnetic resonance imaging (CMR). **Methods:** In this prospective cross-sectional study, 56 young adults with CMR-confirmed BAV and 56 age- and sex-matched healthy volunteers underwent standardized CMR. Biventricular volumes and function, LV global longitudinal strain (LV-GLS), LV torsional mechanics, ascending aortic geometry, pulse-wave velocity, aortic distensibility, and two-dimensional phase-contrast-derived forward and backward flow parameters were quantified. Associations between myocardial mechanics and haemodynamic variables were assessed using correlation analyses and multivariable linear regression. **Results:** Compared with healthy controls, patients with BAV exhibited preserved LV ejection fraction but impaired LV-GLS, reduced LV torsion, increased aortic stiffness, and a significantly greater backward-flow burden despite similar net aortic flow (all *p* < 0.001). Higher peak backward-to-forward flow-rate ratios were associated with less negative LV-GLS (*p* = 0.006), whereas greater aortic regurgitant volume was also associated with impaired longitudinal deformation (*p* = 0.001). In multivariable analysis, LV-GLS and aortic regurgitant volume remained associated with the peak backward-to-forward flow-rate ratio within the specified model, whereas ascending-aortic dimensions were not. The final model explained 52% of the variability in backward-flow burden. **Conclusions:** CMR identified a subclinical ventricular-aortic phenotype in young adults with BAV characterized by altered LV mechanics, increased aortic stiffness, and greater relative backward-flow burden despite preserved conventional LV systolic function. The association between backward-flow burden and LV longitudinal mechanics suggests that these abnormalities may represent interrelated manifestations of BAV disease; however, their temporal and causal relationships cannot be established from this cross-sectional study.

## 1. Introduction

BAV is the most common congenital cardiac malformation, affecting approximately 0.5–2% of the general population [1,2,3]. Although many individuals remain asymptomatic during childhood and early adulthood, BAV is associated with premature aortic stenosis, aortic regurgitation, infective endocarditis, ascending-aortic dilatation, and, less frequently, acute aortic syndromes [1,2,3]. BAV should therefore be regarded as a heterogeneous valvular-aortic disease involving the aortic valve, the left ventricle (LV), and the thoracic aorta rather than as an isolated valvular anomaly [1,2,3]. Current guidelines recommend lifelong clinical and imaging surveillance, with management based primarily on valve dysfunction, symptoms, LV remodelling, aortic dimensions, and the rate of aortic enlargement [1,2,3].

The mechanisms underlying BAV-associated aortopathy are multifactorial and appear to involve both intrinsic abnormalities of the aortic wall and abnormal valve-mediated haemodynamic loading [2,3,4,5]. Asymmetric BAV opening can generate an eccentric systolic jet, abnormal flow directionality, helicity, vortical flow, and non-uniform wall shear stress within the ascending aorta [4,5]. Recent four-dimensional flow cardiovascular magnetic resonance studies have shown that abnormal transvalvular-flow direction and wall shear stress are related to ascending-aortic geometry, while longitudinal observations have associated greater regional wall shear stress exposure with subsequent aortic enlargement [4,5]. These findings support the concept that valve-generated haemodynamic forces may contribute to the regional progression of BAV aortopathy rather than merely accompany established aortic dilatation [4,5].

Reverse aortic flow has recently emerged as another potentially relevant marker of disturbed haemodynamics in BAV [6]. In a 4D-flow CMR study of 655 participants, systolic reverse flow was increased in patients with BAV even in the absence of advanced valve dysfunction and was associated with both aortic stenosis and maximal ascending-aortic (AA) diameter [6]. Reverse-flow burden may reflect complex recirculation and flow separation within the AA and is not necessarily equivalent to conventional diastolic aortic regurgitation [6]. However, the relationships between backward flow, conventional aortic regurgitant volume, aortic geometry, vascular stiffness, and myocardial adaptation remain incompletely characterized [4,6].

Aortic stiffness may represent an additional functional component of BAV-associated aortopathy [7,8]. CMR studies have demonstrated that pulse-wave velocity and AA distensibility vary according to valve function, aortic phenotype, measurement location, and the characteristics of the studied population [7,8]. In a 597-subject 4D-flow CMR study, global aortic pulse-wave velocity was not significantly elevated in patients with normally functioning BAV but was increased in those with concomitant aortic stenosis [7]. Serial CMR evaluation has nevertheless shown a more rapid age-related decline in AA distensibility among patients with BAV, suggesting that functional stiffening may develop before substantial dimensional progression becomes evident [8]. Aortic diameter and stiffness therefore provide related but non-interchangeable information regarding proximal aortic remodelling [7,8].

BAV may also be associated with subclinical LV dysfunction despite preservation of conventional LV ejection fraction [9,10]. Contemporary deformation-imaging studies have identified altered longitudinal shortening, myocardial work, and strain–volume relationships in patients with BAV compared with healthy controls [9,10]. Impaired LV-GLS has been associated with adverse clinical outcomes in BAV independently of preserved ejection fraction, supporting its potential value as a marker of myocardial vulnerability [9,10]. Alterations in ventricular rotation, twist, torsion, and diastolic untwisting may provide additional information concerning the interaction between ventricular loading, myocardial architecture, and valvular–aortic haemodynamics [9,11].

CMR provides an integrated and reproducible method for evaluating ventricular volumes and mass, myocardial deformation, aortic dimensions, vascular stiffness, and time-resolved blood flow within a single examination [6,7,8,9,11]. Cine CMR combined with feature-tracking analysis enables quantification of longitudinal strain and rotational mechanics, whereas phase-contrast CMR permits direct measurement of forward- and backward-flow rates and volumes without geometric assumptions [9,11]. Although 4D-flow CMR offers comprehensive three-dimensional assessment of complex flow patterns and wall shear stress, conventional two-dimensional phase-contrast imaging remains more widely available and can provide clinically accessible measurements of forward flow, backward flow, regurgitant volume, and their temporal characteristics [6,11].

Few studies have simultaneously evaluated myocardial deformation and torsional mechanics, ascending-aortic geometry and stiffness, and forward- and backward-flow characteristics in young adults with BAV [4,6,7,8,9]. Consequently, it remains uncertain how increased backward-flow burden relates to myocardial mechanical abnormalities, conventional ventricular systolic function, aortic size, and valvular regurgitation [6,9,10]. The present study therefore aimed to comprehensively characterize LV deformation and torsional mechanics, AA geometry and stiffness, and two-dimensional phase-contrast CMR-derived forward- and backward-flow parameters in young adults with BAV compared with healthy volunteers [4,6,7,8,9]. We further sought to evaluate clinical, ventricular, and aortic variables associated with the peak backward-to-forward flow-rate ratio [6,7,8,9]. We hypothesized that patients with BAV would demonstrate impaired myocardial deformation, altered torsional mechanics, increased aortic stiffness, and a disproportionate backward-flow burden despite largely preserved conventional LV systolic function.

## 2. Materials and Methods

### 2.1. Study Design and Population

This prospective, single-centre, cross-sectional observational study was conducted at the Department of Internal Medicine, “Iuliu Hatieganu” University of Medicine and Pharmacy, Cluj-Napoca, Romania, between November 2021 and May 2026. Consecutive patients with a transthoracic echocardiographic diagnosis of BAV were screened for inclusion. Participants underwent clinical evaluation, transthoracic echocardiography (TTE), and CMR, with TTE and CMR performed on the same day.

**Figure 1:**
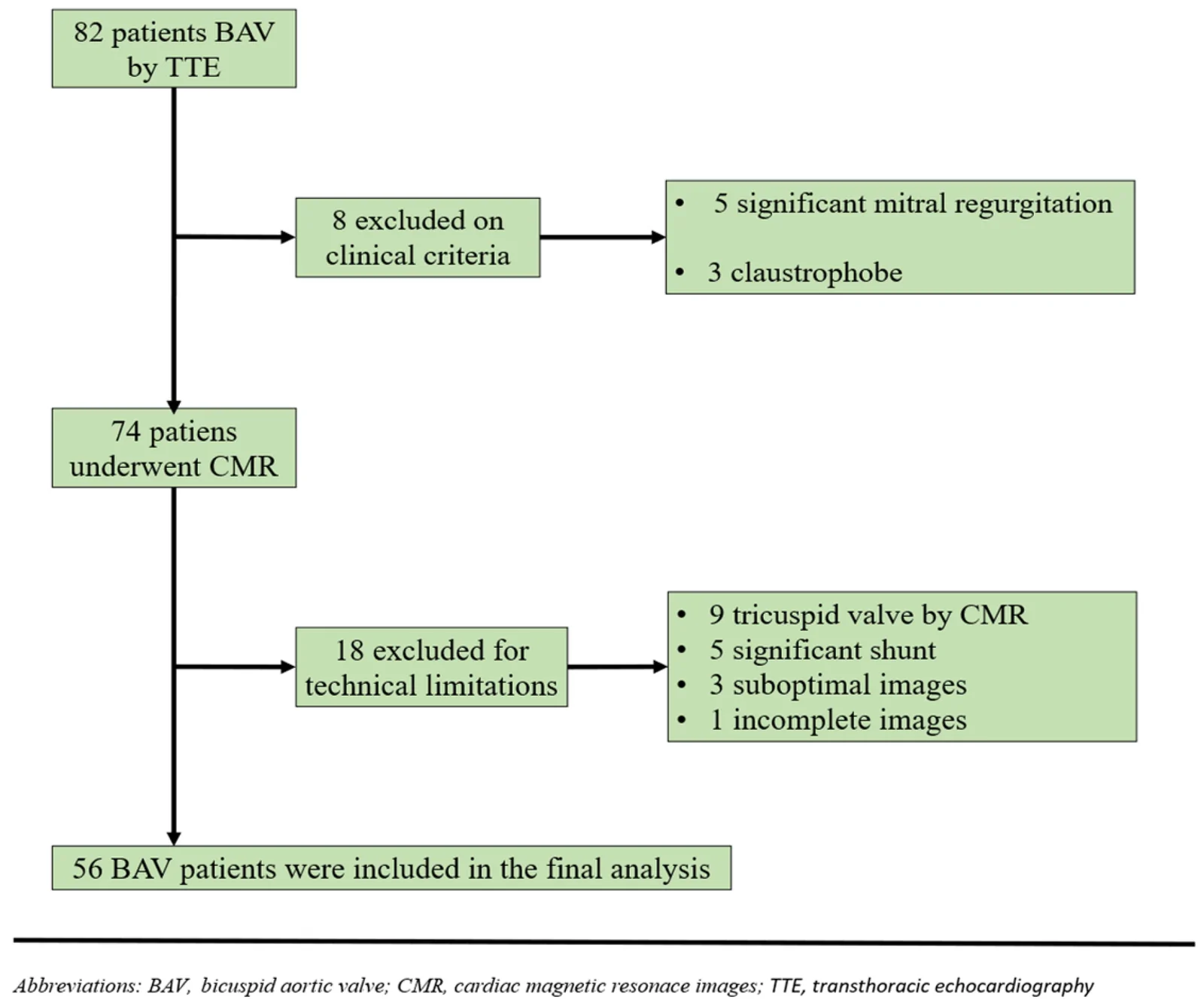
Study flow diagram. A total of 82 patients with a transthoracic echocardiographic diagnosis of bicuspid aortic valve were screened. Eight patients were excluded before cardiovascular magnetic resonance because of significant mitral regurgitation (n = 5) or severe claustrophobia (n = 3). Of the 74 patients who underwent cardiovascular magnetic resonance, 18 were excluded because of a tricuspid aortic valve (n = 9), a haemodynamically significant shunt (n = 5), suboptimal image quality (n = 3), or incomplete image acquisition (n = 1). The final analysis included 56 patients with CMR-confirmed bicuspid aortic valve. BAV, bicuspid aortic valve; CMR, cardiovascular magnetic resonance; TTE, transthoracic echocardiography.

Of 82 patients screened, eight were excluded before CMR because of significant mitral regurgitation (n = 5) or severe claustrophobia (n = 3). The remaining 74 participants underwent CMR. Eighteen were subsequently excluded because CMR demonstrated a tricuspid aortic valve (n = 9), a haemodynamically significant intracardiac or extracardiac shunt (n = 5), suboptimal image quality (n = 3), or incomplete image acquisition (n = 1). The final BAV cohort therefore comprised 56 patients (Figure 1). The control group consisted of 56 healthy volunteers selected to provide a similar age and sex distribution. Cardiovascular health was established from medical history, physical examination, resting 12-lead electrocardiography, TTE, and CMR, with no clinically relevant structural or functional cardiovascular abnormality identified.

Eligibility criteria for the BAV cohort were age ≥ 18 years and CMR-confirmed BAV. Exclusion criteria were a tricuspid aortic valve on CMR, Turner syndrome, a haemodynamically significant systemic-to-pulmonary or pulmonary-to-systemic shunt, significant concomitant mitral regurgitation, a contraindication to CMR, or inadequate image quality for ventricular, myocardial deformation, aortic, or flow analysis. The study protocol was approved by the institutional ethics committee of the “Iuliu Hatieganu” University of Medicine and Pharmacy, Cluj-Napoca, Romania (approval no. 250/30 June 2021). The study was conducted in accordance with the principles of the Declaration of Helsinki and applicable institutional regulations. All participants provided written informed consent before enrolment.

### 2.2. Clinical and Transthoracic Echocardiographic Assessment

Demographic and clinical variables recorded at enrolment included age, sex, height, weight, body mass index, body surface area, systolic and diastolic blood pressure, smoking status, hypertension, and family history of BAV. Brachial blood pressure was measured after an appropriate resting period using a calibrated device.

Comprehensive TTE was performed by experienced investigators according to a standardized protocol using a commercially available system (Vivid E95 GE HealthCare, Horten, Norway). Aortic valve morphology was evaluated in multiple imaging planes. Type 0 BAV was defined by the presence of two cusps without a visible raphe. Type 1 BAV was defined by one raphe and was subclassified according to the cusp-fusion pattern as right–left coronary cusp fusion, right–non-coronary cusp fusion, or left–non-coronary cusp fusion. Peak transaortic velocity and peak and mean transaortic gradients were measured using continuous-wave Doppler. Aortic stenosis and aortic regurgitation were graded using an integrative multiparametric assessment. The aortic root and AA were evaluated, and the examination included screening for coarctation, intracardiac shunts, anomalous pulmonary venous return, and concomitant mitral valve disease.

### 2.3. Cardiovascular Magnetic Resonance Acquisition

All examinations were performed on a 1.5-T scanner (Magnetom Symphony, Siemens Medical Solutions, Erlangen, Germany) using electrocardiographic gating and a dedicated cardiac coil. Breath-held balanced steady-state free-precession cine images were acquired in standard two-, three-, and four-chamber long-axis views and as contiguous short-axis slices from the atrioventricular ring to the apex. These datasets were used for biventricular volumetric analysis, myocardial mass quantification, and CMR feature-tracking analysis.

Two-dimensional phase-contrast CMR with through-plane velocity encoding was performed before contrast administration. After completion of the non-contrast acquisitions, gadobutrol (Gadovist, Bayer AG, Leverkusen, Germany; concentration 1.0 mmol/mL) was administered for the contrast-enhanced components of the institutional CMR protocol. Contrast-enhanced images were not used for the measurements reported in the present study. Images were analyzed by two independent observers, blinded to all clinical and echocardiography data.

### 2.4. Ventricular Volume and Functional Analysis

CMR datasets were analyzed using the cvi42 software (version 6.0.0; Circle Cardiovascular Imaging, Calgary, AB, Canada). The primary observer was blinded to the participants’ clinical and echocardiographic data. LV and right ventricular (RV) endocardial contours were delineated on contiguous short-axis cine images at end-diastole and end-systole. Epicardial contours were delineated at end-diastole for myocardial mass measurements. Semi-automatically generated contours were visually inspected and manually corrected when necessary.

Papillary muscles and trabeculations were assigned to the ventricular blood pool and excluded from volume and ventricular mass measurements. End-diastolic volume, end-systolic volume, stroke volume, ejection fraction, and myocardial mass were calculated for both ventricles. Volumes and myocardial mass were indexed to body surface area.

### 2.5. Left Ventricular Feature-Tracking Analysis

LV myocardial deformation and rotational mechanics were assessed using CMR feature tracking in cvi42. LV-GLS was derived from the two-, three-, and four-chamber cine images. Endocardial and epicardial contours were delineated at end-diastole and propagated throughout the cardiac cycle. Tracking quality was visually evaluated in each view, and contours were manually adjusted when necessary. LV-GLS was defined as peak systolic longitudinal shortening and was reported as a negative percentage, with more negative values indicating greater longitudinal shortening.

LV rotational mechanics were assessed using basal and apical short-axis cine images. The basal slice was defined as the most basal level demonstrating a complete myocardial circumference throughout the cardiac cycle. The apical slice was defined as the most apical level at which the LV cavity and myocardium remained clearly identifiable throughout the cycle.

LV twist was calculated as apical rotation minus basal rotation. LV torsion was calculated by dividing LV twist by the longitudinal distance between the selected basal and apical slices and was expressed in degrees per centimetre. Peak systolic torsion rate and peak diastolic untwisting rate were derived from the temporal torsion curve and expressed in °·cm^−1^·s^−1^. Systolic twisting was assigned a positive value, whereas diastolic untwisting was reported as a negative value.

### 2.6. Ascending Aortic Geometry and Stiffness

Ascending aortic geometry was assessed using cine and phase-contrast images obtained perpendicular to the vessel centreline. Maximum and minimum ascending aortic cross-sectional areas during the cardiac cycle were measured at the predefined analysis plane and indexed to body surface area. The maximal ascending aortic diameter was measured on a double-oblique plane perpendicular to the vessel axis. For descriptive analyses, ascending aortic dilatation was defined as a maximal diameter > 40 mm. Aortic arch length was measured along the vessel centreline between the proximal and distal phase-contrast measurement planes.

AA distensibility was calculated as: Aortic $distensibility=\frac{AAmax-AAmin}{AAmin\times pulse\;pressure}$.

Pulse pressure was calculated as systolic minus diastolic brachial blood pressure. When distensibility was expressed in kPa^−1^, pressure values were converted to kilopascals before calculation.

Pulse-wave velocity was calculated using the transit-time method: $PWV=\frac{\Delta x}{\Delta t}$, where Δx represents the centreline distance between the proximal and distal flow-measurement planes and Δt represents the transit-time delay between the corresponding aortic flow waveforms.

### 2.7. Two-Dimensional Phase-Contrast CMR and Aortic Flow Analysis

Breath-held two-dimensional phase-contrast sequences with through-plane velocity encoding were prescribed perpendicular to aortic flow at the predefined proximal and distal measurement locations.

The acquisition parameters were as follows: velocity-encoding sensitivity, 200 cm/s; field of view, 320–400 × 250–300 mm^2^; acquisition matrix, 256 × 128; slice thickness, 5 mm; flip angle, 25°; repetition time, 5.3 ms; echo time, 3.3 ms; effective temporal resolution, 20 ms; and reconstructed temporal resolution, 10 ms. Each acquisition was reviewed for velocity aliasing, motion artefacts, incomplete vessel coverage, and inadequate lumen delineation.

Phase-contrast datasets were analyzed using cvi42. The aortic lumen was segmented throughout the cardiac cycle using semi-automatic contours, which were manually corrected when necessary. Background phase-offset correction was performed using stationary tissue. Instantaneous volumetric flow was obtained by integrating through-plane velocity over the segmented aortic lumen, generating a time-resolved flow curve.

Flow directed distally along the aorta was defined as forward flow and assigned a positive sign. Flow in the opposite direction was defined as backward flow and assigned a negative sign.

In the present study, the term backward flow was used as a directional phase-contrast CMR descriptor and was not considered synonymous with aortic regurgitation. Negative flow may occur during different phases of the cardiac cycle. Negative flow occurring during systole may reflect flow separation, recirculation, or complex secondary flow within the ascending aorta, whereas diastolic negative flow includes conventional valvular aortic regurgitation. Because two-dimensional phase-contrast CMR does not provide full three-dimensional characterization of vortical or helical flow, these mechanisms should be regarded as physiologically plausible interpretations rather than directly measured flow patterns.

Peak forward-flow rate (Q_FFmax_) was defined as the maximum positive flow rate. Peak backward-flow rate (Q_BFmax_) was reported as the absolute magnitude of the most negative flow rate. Forward-flow volume (V_FF_) and total backward-flow volume (V_BF_) were calculated by integrating the positive and negative components of the flow curve, respectively, over the complete cardiac cycle. V_BF_ therefore represented the total magnitude of negative flow detected at the measurement plane, irrespective of its timing within the cardiac cycle, and was reported as an absolute positive magnitude.

Net flow volume was calculated as: Vnet=VFF−VBF. Aortic regurgitant volume (V_Reg_) was quantified separately from the diastolic reverse-flow component at the proximal ascending aortic measurement plane. Regurgitant fraction was calculated as: $Regurgitant\;fraction=\frac{Vreg}{VFF}\times 100$.

In contrast, aortic regurgitant volume (V_Reg_) represented the diastolic reverse-flow component attributable to valvular aortic regurgitation at the proximal ascending-aortic measurement plane. Accordingly, V_BF_ and V_Reg_ were considered related but non-interchangeable haemodynamic parameters.

Backward-flow appearance time was defined as the interval from the beginning of the cardiac cycle to the first sustained occurrence of negative flow. Time to peak backward flow was measured from the same temporal origin.

Relative backward-flow burden was expressed as Q_BFmax_/Q_FFmax_ × 100 and V_BF_/V_FF_ × 100.

The Q_BFmax_/Q_FFmax_ ratio was selected as the principal relative backward-flow index because it expresses the magnitude of the maximal instantaneous reverse-flow event relative to the individual’s maximal antegrade-flow rate and therefore reduces the influence of interindividual differences in absolute flow magnitude. In contrast, V_BF_/V_FF_ reflects the integrated volumetric reverse-flow burden and is additionally influenced by the duration of negative flow. Q_BFmax_/Q_FFmax_ should therefore be interpreted as a measure of relative reverse-flow intensity rather than as a specific measure of systolic recirculation or aortic regurgitation.

**Figure 2:**
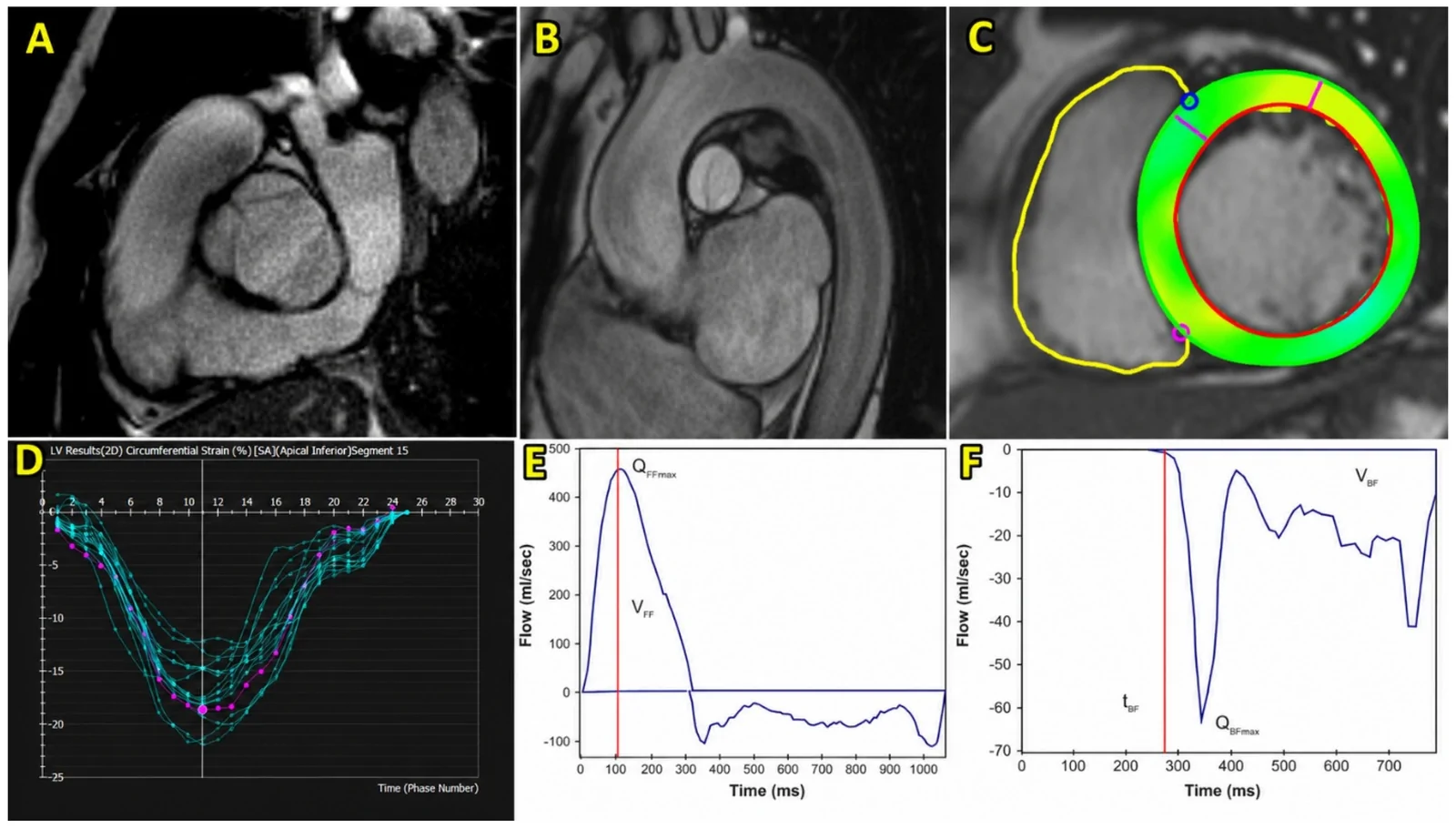
Representative cardiovascular magnetic resonance acquisition and analysis workflow. (**A**) Cine CMR image showing the left ventricular outflow tract, aortic valve, and proximal ascending aorta. (**B**) Oblique sagittal image of the thoracic aorta used for positioning the phase-contrast acquisition plane perpendicular to the direction of aortic flow. (**C**) Representative short-axis feature-tracking segmentation, including LV endocardial and epicardial contours. (**D**) Representative myocardial strain curves generated throughout the cardiac cycle. (**E**) Complete phase-contrast flow–time curve showing the positive forward-flow component and the negative backward-flow component. (**F**) Isolated negative-flow component used to derive peak backward-flow rate, backward-flow volume, backward-flow appearance time, and time to peak backward flow. CMR, cardiovascular magnetic resonance; LV, left ventricle.

Representative CMR acquisition planes, feature-tracking analysis, and the derivation of forward- and backward-flow parameters are illustrated in Figure 2.

### 2.8. Reproducibility Analysis

Intra-observer and inter-observer reproducibility were evaluated by repeated analyses in all 56. For intra-observer reproducibility, the primary observer repeated the measurements after a prespecified interval while blinded to the initial results. For inter-observer reproducibility, a second independent observer, blinded to the first observer’s measurements and to the participants’ clinical data, repeated the analyses.

Reproducibility was evaluated for LV-GLS, LV torsion, minimum AA area, aortic regurgitant volume, and backward-flow volume. Agreement was quantified using intraclass correlation coefficients derived from a two-way absolute-agreement model and reported with 95% confidence intervals.

### 2.9. Statistical Analysis

Statistical analyses were performed using MedCalc Statistical Software, version 23.4.5 (MedCalc Software Ltd., Ostend, Belgium). The distribution of continuous variables was assessed using the Shapiro–Wilk test together with visual inspection of histograms and quantile–quantile plots. Normally distributed variables were summarized as mean ± standard deviation, whereas non-normally distributed variables were reported as median with range or interquartile range, as appropriate. Categorical variables were reported as number and percentage.

Comparisons between patients with BAV and healthy volunteers were performed using the independent-samples Student’s *t*-test for normally distributed continuous variables and the Mann–Whitney U-test for non-normally distributed variables. Categorical variables were compared using the chi-square test or Fisher’s exact test, as appropriate.

Associations between continuous variables were evaluated using Pearson or Spearman correlation coefficients according to variable distribution and the form of the relationship. Because all measurements were obtained at a single study visit, associations with age were interpreted as cross-sectional age-associated differences and not as longitudinal changes.

Multivariable linear regression analysis was performed to evaluate variables associated with the Q_BFmax_/Q_FFmax_ ratio. Four models were evaluated: Model A included age, sex, and body surface area. Model B included the variables from Model A together with indexed maximum AA area, indexed minimum AA area, and aortic regurgitant volume. Model C included the variables from Model A together with LV-GLS and LV torsion. Model D included the variables from Model A together with aortic regurgitant volume and LV-GLS.

The regression models were designed as exploratory, parsimonious models rather than exhaustive predictive models. The number of covariates was deliberately limited because of the modest size of the BAV cohort and the expected interdependence among candidate haemodynamic variables. Peak aortic velocity and transaortic gradients represent closely related measures of valvular obstruction; maximum diameter and indexed aortic areas describe overlapping aspects of aortic geometry; and pulse-wave velocity, distensibility, and blood pressure reflect partially overlapping components of arterial load. Simultaneous inclusion of a large number of these variables was avoided to reduce model instability and multicollinearity. Accordingly, the associations identified should be interpreted within the specified models rather than as evidence that the retained variables are the only independent determinants of backward-flow burden.

Regression coefficients, standard errors, and individual *p*-values were reported. Overall model performance was summarized using R^2^. Model assumptions were evaluated by examining linearity, residual normality, homoscedasticity, influential observations, and multicollinearity.

Complete-case analysis was used for variables with missing data, and no values were imputed. All statistical tests were two-sided, and a *p*-value < 0.05 was considered statistically significant. No formal adjustment for multiple comparisons was applied because several analyses were exploratory. Consequently, nominal *p*-values from secondary analyses should be interpreted cautiously and in conjunction with effect size, biological plausibility, and consistency across related findings rather than as confirmatory evidence.

## 3. Results

### 3.1. Study Population and Baseline Characteristics

A total of 82 patients with a transthoracic echocardiographic diagnosis of bicuspid aortic valve were screened for eligibility. Eight patients were excluded before cardiovascular magnetic resonance examination because of clinically significant mitral regurgitation (n = 5) or severe claustrophobia precluding CMR (n = 3). The remaining 74 patients underwent CMR. Eighteen additional patients were excluded after CMR because of a tricuspid aortic valve (n = 9), a haemodynamically significant intracardiac or extracardiac shunt (n = 5), suboptimal image quality (n = 3), or incomplete image acquisition (n = 1). The final BAV cohort therefore comprised 56 patients.

The control group comprised 56 healthy volunteers. Patients with BAV and healthy volunteers had similar age and sex distributions. The median age was 25 years in both groups, with a reported range of 18–33 years in the BAV group and 17–32 years in the control group (*p* = 0.536). The BAV cohort included 35 men and 21 women, compared with 36 men and 20 women in the control group.

Body surface area, body mass index, systolic blood pressure, and diastolic blood pressure did not differ significantly between groups. Smoking prevalence was also similar, with 11 smokers (19.6%) in the BAV group and 13 (23.2%) among healthy volunteers (*p* = 0.610). Within the BAV cohort, 26 patients (46.4%) had hypertension and nine (16.1%) reported a family history of BAV.

Transaortic haemodynamic parameters were higher in patients with BAV. Peak aortic velocity was 2.06 ± 1.12 m/s in patients with BAV compared with 1.34 ± 0.12 m/s in controls (*p* = 0.001). Peak and mean transaortic gradients were also higher in the BAV group (19.2 ± 8.42 vs. 7.15 ± 2.14 mmHg and 9.8 ± 6.34 vs. 3.6 ± 0.79 mmHg, respectively; both *p* = 0.001).

Type 0 BAV morphology was identified in 22 patients (39.2%), whereas 34 patients had type 1 morphology. Among patients with type 1 BAV, right–left coronary cusp fusion was present in 21, right–non-coronary cusp fusion in nine, and left–non-coronary cusp fusion in four.

Ascending-aortic dilatation, defined as a maximal diameter > 40 mm, was present in 21 patients (37.5%). Of these, eight had a root-predominant phenotype and 13 had an ascending-aorta-predominant phenotype.

Aortic regurgitation was absent in 39 patients (69.6%), mild in 12 (21.4%), and moderate in five (8.9%). None had severe aortic regurgitation. Aortic stenosis was present in nine patients, including six with mild stenosis and three with moderate stenosis. No patient had coarctation of the aorta.

### 3.2. Biventricular Volumes and Systolic Function

CMR-derived ventricular parameters are presented in Table 1. LV end-diastolic volume index was 74.1 ± 13.3 mL/m^2^ in patients with BAV and 68.1 ± 16.7 mL/m^2^ in healthy volunteers (*p* = 0.050). LV end-systolic volume index did not differ significantly between groups (25.7 ± 8.1 vs. 24.3 ± 7.5 mL/m^2^; *p* = 0.312).

LV stroke volume index was higher in the BAV group than in controls (48.3 ± 8.1 vs. 43.9 ± 11.5 mL/m^2^; *p* = 0.035). Despite this difference, LV ejection fraction was similar between groups (65.7 ± 7.7% vs. 65.8 ± 6.0%; *p* = 0.343). Indexed LV mass was higher in patients with BAV (62.6 ± 9.9 vs. 57.5 ± 14.8 g/m^2^; *p* = 0.029).

RV end-diastolic volume index did not differ significantly between patients with BAV and controls (71.6 ± 17.1 vs. 66.5 ± 17.3 mL/m^2^; *p* = 0.115). In contrast, RV end-systolic volume index was higher in the BAV group (33.5 ± 14.8 vs. 25.2 ± 8.0 mL/m^2^; *p* < 0.001).

RV stroke volume index was numerically lower in patients with BAV, although the difference did not reach statistical significance (37.9 ± 9.1 vs. 41.3 ± 11.4 mL/m^2^; *p* = 0.054). RV ejection fraction was lower in patients with BAV than in healthy volunteers (54.2 ± 12.2% vs. 62.1 ± 6.1%; *p* < 0.001). Indexed RV mass did not differ significantly between groups (56.2 ± 6.9 vs. 50.8 ± 3.7 g/m^2^; *p* = 0.119).

### 3.3. Left Ventricular Deformation and Torsional Mechanics

Despite similar conventional LV ejection fractions, patients with BAV demonstrated significant differences in myocardial deformation and rotational mechanics (Table 1).

LV global longitudinal strain was less negative in patients with BAV than in healthy volunteers (−19.7 ± 1.31% vs. −21.2 ± 1.92%; *p* < 0.001), indicating a lower absolute magnitude of longitudinal shortening.

Global LV torsion was also lower in patients with BAV (1.59 ± 0.22 vs. 1.96 ± 0.27°/cm; *p* < 0.001). Conversely, peak systolic LV torsion rate was higher in the BAV group (13.73 ± 0.91 vs. 12.25 ± 1.05°·cm^−1^·s^−1^; *p* < 0.001).

Peak diastolic LV torsion rate was more negative in patients with BAV than in healthy volunteers (−12.64 ± 1.04 vs. −11.61 ± 1.12°·cm^−1^·s^−1^; *p* < 0.001).

### 3.4. Ascending-Aortic Dimensions and Geometry

The maximal ascending-aortic diameter was greater in patients with BAV than in healthy volunteers, with reported values of 34.2 mm (range, 25.1–49.3 mm) and 26.2 mm (range, 24.5–31.2 mm), respectively (*p* < 0.001).

The indexed maximum ascending-aortic area was numerically greater in patients with BAV, although the difference did not reach statistical significance (4.54 ± 1.11 vs. 4.14 ± 1.19 cm^2^/m^2^; *p* = 0.072). The indexed minimum ascending-aortic area was significantly greater in patients with BAV (4.02 ± 1.09 vs. 3.44 ± 1.16 cm^2^/m^2^; *p* = 0.008).

Aortic arch length was 125 ± 20.9 mm in patients with BAV and 118 ± 18.2 mm in controls, without a statistically significant between-group difference (*p* = 0.068).

### 3.5. Aortic Stiffness and Pulsatile Characteristics

Pulse-wave velocity was higher in patients with BAV than in healthy volunteers (6.25 ± 1.54 vs. 5.24 ± 1.57 m/s; *p* = 0.003), whereas ascending-aortic distensibility was lower in the BAV group (2.72 ± 1.10 vs. 5.50 ± 1.50 × 10^−3^ kPa^−1^; *p* < 0.001). Together, these directionally concordant findings indicated impaired aortic elastic properties.

### 3.6. Ascending-Aortic Forward and Backward Flow

Phase-contrast CMR-derived flow parameters are summarized in Table 2. Global aortic net-flow volume did not differ significantly between patients with BAV and healthy volunteers (59.69 ± 17.0 vs. 62.04 ± 19.1 mL; *p* = 0.494).

Aortic regurgitant volume was markedly higher in the BAV group (13.17 ± 8.05 vs. 0.71 ± 0.57 mL; *p* < 0.001). The corresponding regurgitant fraction was also higher in patients with BAV (13.75 ± 8.39% vs. 0.33 ± 0.08%; *p* < 0.001).

Peak backward-flow rate was higher in patients with BAV than in healthy volunteers (51.47 ± 21.8 vs. 43.87 ± 20.2 mL/s; *p* < 0.001). Total backward-flow volume was likewise greater in the BAV group (16.70 ± 11.4 vs. 9.59 ± 4.4 mL; *p* = 0.001).

Backward-flow appearance time did not differ significantly between groups (147 ± 73.7 vs. 166 ± 79.8 ms; *p* = 0.052). Time to peak backward flow was also comparable (283 ± 75.6 vs. 303 ± 59.4 ms; *p* = 0.057).

Peak forward-flow rate was lower in patients with BAV than in healthy volunteers (238.42 ± 73.9 vs. 297.71 ± 85.4 mL/s; *p* < 0.001). In contrast, forward-flow volume was higher in patients with BAV (75.84 ± 20.1 vs. 70.87 ± 22.3 mL; *p* = 0.027).

Both indices expressing backward-flow burden relative to forward flow were higher in patients with BAV. The peak backward-to-forward flow-rate ratio was 21.40 ± 9.2% in patients with BAV and 14.49 ± 6.3% in controls (*p* < 0.001). Similarly, the backward-to-forward flow-volume ratio was higher in the BAV group (22.02 ± 18.7% vs. 13.60 ± 8.2%; *p* < 0.001).

### 3.7. Associations Between LV Longitudinal Deformation and Aortic Parameters

**Figure 3:**
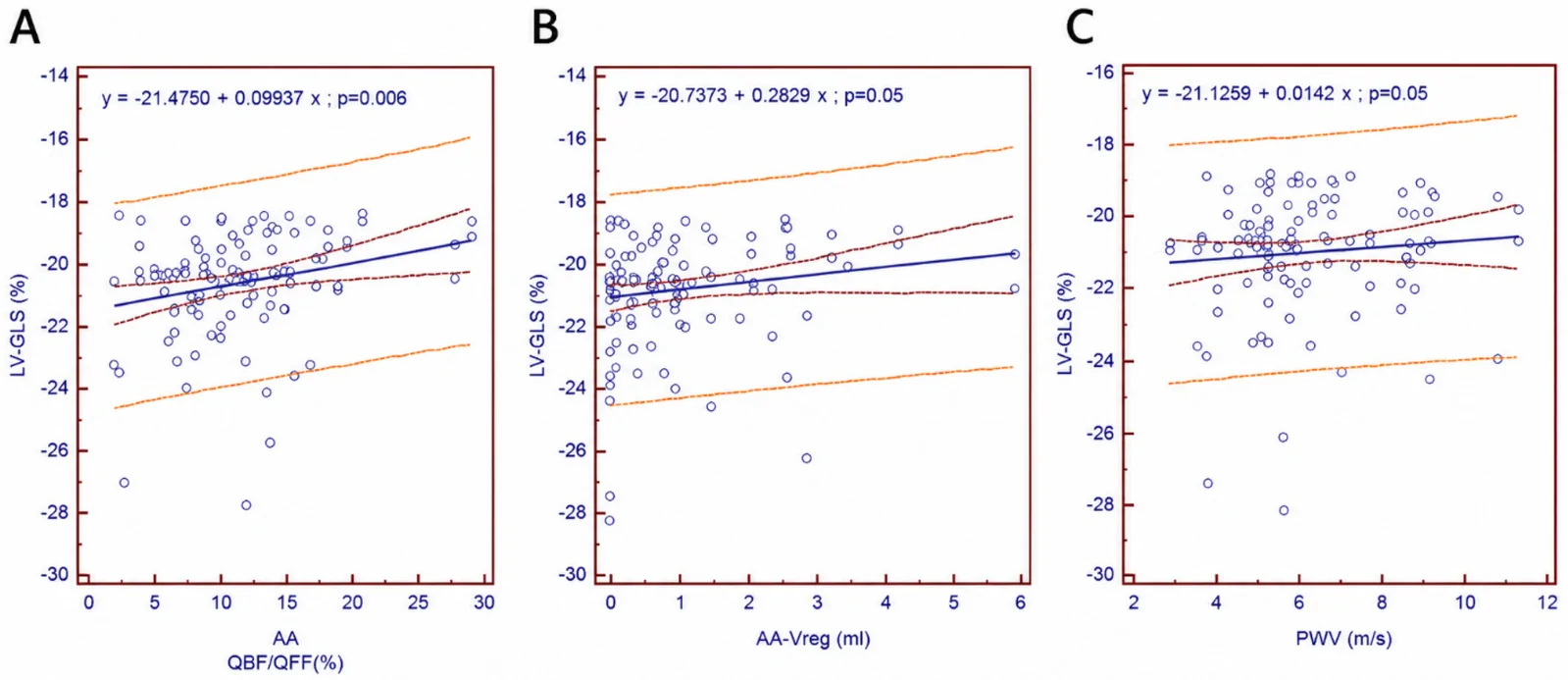
Associations between left ventricular global longitudinal strain and ascending aortic haemodynamic and stiffness parameters. Scatterplots demonstrate the relationships of LV-GLS with (**A**) the peak backward-to-forward flow-rate ratio, Q_BFmax_/Q_FFmax_; (**B**) aortic regurgitant volume; and (**C**) pulse-wave velocity. Solid lines represent the fitted linear regression lines, inner dashed lines represent the 95% confidence intervals, and outer dashed lines represent the prediction intervals. LV-GLS, left ventricular global longitudinal strain; PWV, pulse-wave velocity; Q_BFmax_, peak backward-flow rate; Q_FFmax_, peak forward-flow rate.

Higher peak backward-to-forward flow-rate ratios were associated with progressively less negative LV-GLS values (*p* = 0.006; Figure 3A). Thus, a greater relative backward-flow burden was associated with a lower absolute magnitude of LV longitudinal shortening.

Aortic regurgitant volume was also positively associated with LV-GLS, with greater regurgitant volumes corresponding to less negative strain values (*p* = 0.001; Figure 3B).

The association between pulse-wave velocity and LV-GLS was weaker and was of borderline statistical significance (*p* = 0.050; Figure 3C).

### 3.8. Associations Between Longitudinal Deformation and Left Ventricular Torsional Mechanics

**Figure 4:**
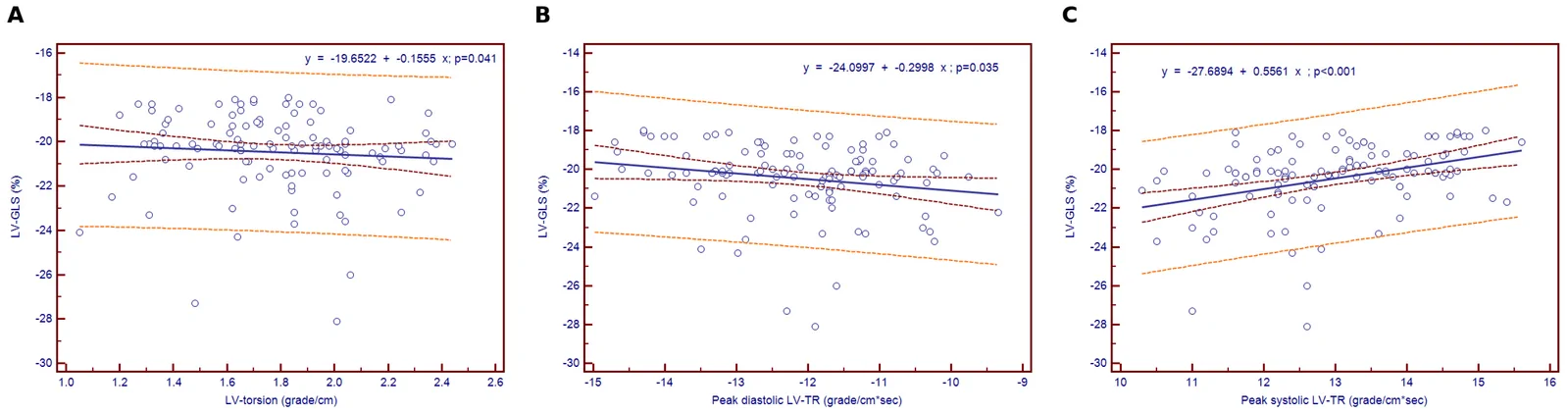
Associations between left ventricular global longitudinal strain and torsional mechanics. Scatterplots demonstrate the relationships of LV global longitudinal strain with (**A**) global LV torsion, (**B**) peak diastolic LV torsion rate, and (**C**) peak systolic LV torsion rate. Solid lines represent the fitted linear regression lines, inner dashed lines represent the 95% confidence intervals, and outer dashed lines represent the prediction intervals. LV, left ventricular; LV-GLS, left ventricular global longitudinal strain.

LV-GLS was significantly associated with all assessed indices of LV torsional mechanics. Higher global LV torsion was associated with more negative LV-GLS values (*p* = 0.041; Figure 4A). LV-GLS was also associated with peak diastolic torsion rate (*p* = 0.035; Figure 4B). The strongest relationship was observed between LV-GLS and peak systolic torsion rate (*p* < 0.001; Figure 4C).

### 3.9. Multivariable Associations with Backward Flow

The multivariable linear regression models evaluating the peak backward-to-forward flow-rate ratio are presented in Table 3.

Model A, which included age, sex, and body surface area, explained 21% of the variability in the Q_BFmax_/Q_FFmax_ ratio and was statistically significant overall (R^2^ = 0.21; *p* < 0.001). Sex was associated with the flow-rate ratio (β = −4.091 ± 1.013; *p* < 0.001), whereas age (*p* = 0.062) and body surface area (*p* = 0.397) were not. After the addition of indexed maximum and minimum ascending-aortic areas and aortic regurgitant volume, Model B explained 43% of the outcome variability (R^2^ = 0.43; overall *p* < 0.001). Age (β = −0.268 ± 0.117; *p* = 0.0245), sex (β = −4.096 ± 1.018; *p* < 0.001), and aortic regurgitant volume (β = 0.113 ± 0.048; *p* = 0.0021) were associated with the Q.

Q_BFmax_/Q_FFmax_ ratio. Body surface area and indexed maximum and minimum ascending-aortic areas were not significant.

Model C, which incorporated LV-GLS and global LV torsion in addition to age, sex, and body surface area, explained 49% of the variability in the flow-rate ratio (R^2^ = 0.49; overall *p* < 0.001). Sex, LV-GLS (β = 0.517 ± 0.049; *p* = 0.0046), and LV torsion (β = 2.966 ± 1.488; *p* = 0.0488) were associated with the outcome, whereas age and body surface area were not.

Model D provided the highest model fit, explaining 52% of the variability in the Q_BFmax_/Q_FFmax_ ratio (R^2^ = 0.52; overall *p* < 0.001). Within Model D, age (β = −0.248 ± 0.111; *p* = 0.0387), sex (β = −4.126 ± 0.966; *p* < 0.001), aortic regurgitant volume (β = 0.173 ± 0.049; *p* = 0.0381), and LV-GLS (β = 0.469 ± 0.229; *p* = 0.0271) were independently associated with the flow-rate ratio. Body surface area was not significant.

Given the modest cohort size and the limited number of covariates that could be included simultaneously, these models should be regarded as exploratory. Potential residual confounding by hypertension, transaortic haemodynamics, valve morphology, aortic stiffness, and other measures of aortic geometry cannot be excluded.

### 3.10. Reproducibility

CMR-derived myocardial deformation, aortic geometry, and flow measurements demonstrated good-to-excellent intra-observer and inter-observer reproducibility.

Intra-observer intraclass correlation coefficients were 0.91 (95% CI, 0.83–0.92) for LV-GLS, 0.93 (95% CI, 0.90–0.95) for LV torsion, 0.95 (95% CI, 0.92–0.96) for minimum ascending-aortic area, 0.94 (95% CI, 0.92–0.95) for aortic regurgitant volume, and 0.92 (95% CI, 0.89–0.93) for backward-flow volume.

The corresponding inter-observer intraclass correlation coefficients were 0.89 (95% CI, 0.75–0.95), 0.88 (95% CI, 0.78–0.94), 0.90 (95% CI, 0.82–0.95), 0.93 (95% CI, 0.85–0.96), and 0.92 (95% CI, 0.80–0.95), respectively.

## 4. Discussion

The present study identified a subclinical ventricular–aortic phenotype in young adults with BAV, characterized by impaired LV longitudinal deformation and torsional mechanics, increased aortic stiffness, and a disproportionate backward-flow burden despite preserved LV ejection fraction [10,12,13,14]. Previous imaging studies have similarly demonstrated that myocardial mechanical abnormalities may be detectable in patients with apparently preserved conventional systolic function and limited valve dysfunction [12,13,14].

These findings reinforce the concept that ejection fraction alone may incompletely characterize ventricular adaptation to the chronic haemodynamic effects of BAV [10,12]. Nevertheless, because myocardial deformation is influenced by preload, afterload, ventricular geometry, and valve dysfunction, the observed abnormalities cannot be interpreted as evidence of a primary BAV-related cardiomyopathy [10,13,14,15].

LV-GLS was less negative in patients with BAV, indicating reduced longitudinal shortening despite an almost identical LV ejection fraction in the two groups [12,13,14]. Earlier CMR feature-tracking and echocardiographic studies have reported impaired longitudinal or multidirectional deformation in clinically normal BAV and across different patterns of valvular dysfunction [12,13,14]. Impaired LV-GLS has also been associated with adverse outcomes in patients with BAV and preserved ejection fraction, supporting its potential relevance as a marker of subclinical myocardial vulnerability [10]. The lower global LV torsion and altered systolic twisting and diastolic untwisting rates observed in our cohort suggest that rotational mechanics may provide complementary information regarding myocardial adaptation [13,15]. However, the coexistence of higher LV stroke volume and indexed mass indicates that altered loading conditions may partly explain these findings [15,16].

The increase in absolute backward-flow volume and in the Q_BFmax_/Q_FFmax_ and V_BF_/_FF_ ratios represents the most distinctive haemodynamic finding of this study [17,18,19,20]. Conventional phase-contrast CMR studies have previously shown that clinically accessible two-dimensional flow measurements can detect abnormal systolic flow patterns related to BAV-associated aortopathy [17]. More comprehensive three-dimensional analyses have demonstrated that backward velocity, flow eccentricity, regurgitant fraction, vorticity, and circumferential wall shear stress can distinguish patients with BAV from healthy individuals even before aortic dilatation becomes evident [18]. Abnormal flow asymmetry and vortex formation are also related to leaflet-fusion characteristics, indicating that valve morphology influences the haemodynamic environment of the ascending aorta [19]. Total backward flow should therefore not be equated with conventional diastolic aortic regurgitation; it may include contributions from flow separation, recirculation, and secondary flow, although these mechanisms were not directly characterized by the two-dimensional acquisition [17,18,19,20].

The relationship between a greater backward-to-forward flow-rate ratio and less negative LV-GLS suggests that abnormal aortic flow and myocardial deformation may form part of a coupled ventricular-aortic phenotype [16,18,21]. An association between arterial stiffness and altered myocardial work has been reported even in isolated BAV, supporting a potential interaction between proximal aortic mechanical properties and LV performance [16]. CMR studies in stenotic BAV have likewise linked reduced aortic distensibility with LV remodelling and impaired myocardial strain despite preserved ejection fraction [21]. The direction of this association cannot be determined from the present cross-sectional data. Greater backward-flow burden may contribute to altered ventricular loading, differences in ventricular ejection may themselves influence aortic reverse-flow patterns, or both findings may arise from shared valvular and aortic abnormalities. The present data therefore demonstrate coexistence and statistical association rather than a temporal or causal relationship [18,21].

The present BAV cohort was haemodynamically heterogeneous and included patients with varying degrees of aortic regurgitation, aortic stenosis, and aortic dilatation. Although most participants had absent or mild valve dysfunction and no patient had severe aortic stenosis or regurgitation, these abnormalities may independently influence both aortic flow patterns and LV mechanics. The small numbers of patients with moderate valve dysfunction and within individual aortic phenotypes precluded reliable inferential subgroup modelling. Consequently, the observed association between backward-flow burden and LV-GLS should not be interpreted as demonstrating a flow phenotype that is fully independent of conventional valvular loading or aortic phenotype. Hypertension was also common in the BAV cohort and was not included in the final multivariable model; residual confounding related to chronic arterial load therefore cannot be excluded despite similar blood-pressure values at the study examination.

Higher PWV and lower ascending-aortic distensibility in the BAV group are consistent with impaired aortic elastic function [22,23,24,25]. CMR studies have shown that aortic stiffness in BAV is heterogeneous and varies according to aortic dimensions, valve phenotype, measurement level, and associated valve dysfunction [22,23]. Regional stiffness may also differ substantially between patients with BAV, Marfan syndrome, and degenerative aortic aneurysms of comparable diameter, suggesting that aortic size alone does not fully describe vessel-wall function [24]. Importantly, CMR-derived distensibility has shown potential prognostic value for subsequent prophylactic valve or aortic surgery, although current evidence is insufficient to define clinically applicable thresholds [25].

The observed haemodynamic abnormalities are biologically compatible with contemporary evidence linking altered flow to regional aortic remodelling [5,26,27,28]. Prospective studies have associated regional wall shear stress and wall shear stress angle with subsequent ascending-aortic growth in BAV [26,27]. Longer-term observations have also demonstrated persistence or evolution of abnormal wall shear stress patterns over extended follow-up [27]. Conversely, low or oscillatory wall shear stress has not consistently correlated with existing aortic dilatation, illustrating that different haemodynamic metrics may reflect distinct biological processes [28]. These results support further investigation of backward-flow indices as accessible complementary markers but do not justify their use as independent clinical intervention thresholds.

The principal limitations are the single-centre design, modest sample size, young and selected population, heterogeneous valve and aortic phenotypes, and cross-sectional assessment. Two-dimensional phase-contrast CMR provides practical quantitative flow measurements but cannot characterize the full three-dimensional distribution of helicity, vortices, flow displacement, and regional wall shear stress available from 4D-flow imaging [17,18,19,20]. The absence of longitudinal imaging and clinical outcomes prevents determination of whether LV-GLS, torsion, stiffness, or backward-flow ratios predict aortic enlargement, valve progression, or cardiovascular events [5,10,25,26,27]. The modest cohort size also limited the complexity of multivariable modelling. Inclusion of a large number of interrelated haemodynamic covariates would have increased the risk of unstable estimates, multicollinearity, and model overfitting; the regression models should therefore be regarded as exploratory and hypothesis-generating. Not all potentially relevant confounders, including hypertension, transaortic haemodynamics, BAV morphology, aortic stiffness, and alternative measures of aortic geometry, were simultaneously incorporated into the models, and residual confounding remains possible. The small number of patients with moderate valve dysfunction and within individual aortic phenotypes precluded adequately powered subgroup analyses. Finally, no formal correction for multiple comparisons was applied; secondary associations should be interpreted cautiously, and the models have not been externally validated.

Overall, the findings suggest that young adults with BAV may exhibit a subclinical ventricular–aortic phenotype despite preserved conventional LV systolic function. Prospective multicentre studies are required to determine whether combined deformation, stiffness, and backward-flow assessment improves risk stratification beyond established valve and aortic measurements.

## 5. Conclusions

In young adults with bicuspid aortic valve, cardiovascular magnetic resonance identified a subclinical ventricular–aortic phenotype characterized by impaired LV longitudinal and torsional mechanics, increased aortic stiffness, and greater relative backward-flow burden despite preserved conventional LV ejection fraction. The association between Q_BFmax_/Q_FFmax_, LV-GLS, and aortic regurgitant volume indicates that altered aortic haemodynamics and myocardial mechanics coexist in BAV; however, the present cross-sectional data cannot establish their temporal sequence or causal relationship.

These findings should not be interpreted as establishing backward flow as an independent BAV-specific mechanism or as an indication for altered clinical management. The modest sample size, heterogeneous valve and aortic phenotypes, and potential residual confounding require that the multivariable findings be considered exploratory. Larger longitudinal studies are needed to determine whether backward-flow indices provide incremental information beyond established measures of valve dysfunction, ventricular remodelling, aortic stiffness, and aortic dimensions.

## Figures and Tables

**Table 1:** Baseline characteristics.

Demographic Characteristics	BAV Patients (n = 56)	Healthy Volunteers (n = 56)	*p*-Value
- Age, years	25 (18–33)	25 (17–32)	0.536
- Male/female, n	35/21	36/20	NS
- Body surface area, m^2^	1.74 (0.2)	1.80 (0.2)	0.056
- Body-mass index, kg/m^2^	23.7 (4.6)	23.9 (3.7)	0.829
- Systolic artery pressure, mmHg	119 (11.8)	115 (11.7)	0.319
- Diastolic artery pressure, mmHg	69 (9.6)	70 (8.3)	0.493
- Hypertension, n (%)	26 (46.4)	-	NA
- Smokers, n (%)	11 (19.6)	13 (23.2)	0.610
- Familial history of BAV, n (%)	9 (16.1)	-	NA
**TTE characteristics**			
- Peak aortic velocity, m/s	2.06 (1.12)	1.34 (0.12)	0.001
- Peak transaortic gradient, mmHg	19.2 (8.42)	7.15 (2.14)	0.001
- Mean transaortic gradient, mmHg	9.8 (6.34)	3.6 (0.79)	0.001
- BAV morphology			
Type 0, n (%)	22 (39.2)	NA	-
Type 1 (RL, RN, LN), n	34 (21/9/4)	NA	-
- Dilated ascending aorta (>40 mm), n (%)	21 (37.5)	NA	-
- Root/ascending phenotype, n	8/13	NA	-
- Aortic regurgitation grade			
None, n (%)	39 (69.6)	NA	-
Mild, n (%)	12 (21.4)	NA	-
Moderate, n (%)	5 (8.9)	NA	-
- Aortic stenosis			
Mild, n (%)	6 (10.7)	NA	-
Moderate, n (%)	3 (5.3)	NA	-
**CMR characteristics**			
- LV end-diastolic volume indexed, mL/m^2^	74.1 (13.3)	68.1 (16.7)	0.050
- LV end-systolic volume indexed, mL/m^2^	25.7 (8.1)	24.3 (7.5)	0.312
- LV stroke volume indexed, mL/m^2^	48.3 (8.1)	43.9 (11.5)	0.035
- LV ejection fraction, %	65.7 (7.7)	65.8 (6.0)	0.343
- LV mass indexed, g/m^2^	62.6 (9.9)	57.5 (14.8)	0.029
- LV-GLS, %	−19.7 (1.31)	−21.2 (1.92)	<0.001
- LV-torsion (grade/cm)	1.59 (0.22)	1.96 (0.27)	<0.001
- Peak systolic LV-TR (grade/cm*sec)	13.73 (0.91)	12.25 (1.05)	<0.001
- Peak diastolic LV-TR (grade/cm*sec)	−12.64 (1.04)	−11.61 (1.12)	<0.001
- RV end-diastolic volume indexed, mL/m^2^	71.6 (17.1)	66.5 (17.3)	0.115
- RV end-systolic volume indexed, mL/m^2^	33.5 (14.8)	25.2 (8.0)	<0.001
- RV stroke volume indexed, mL/m^2^	37.9 (9.1)	41.3 (11.4)	0.054
- RV ejection fraction, %	54.2 (12.2)	62.1 (6.1)	<0.001
- RV mass indexed, g/m^2^	56.2 (6.9)	50.8 (3.7)	0.119
- Maximal aortic diameter, mm	34.2 (25.1–49.3)	26.2 (24.5–31.2)	<0.001

Abbreviations: BAV, bicuspid aortic valve; CMR, cardiovascular magnetic resonance; LV, left ventricle; LV-GLS, left ventricular global longitudinal strain; LV-TR, left ventricular torsion rate; TTE, transthoracic echocardiography; RL, right-left coronary cusp fusion; RN, right-noncoronary cusp fusion; LN, left-noncoronary cusp fusion; RV, right ventricle.

**Table 2:** Ascending-aortic geometry, stiffness, and flow measurements in patients with BAV and healthy volunteers.

Variables	BAV Patients (n = 56)	Healthy Volunteers (n = 56)	*p*-Value
**Aortic geometry**			
Area AA_max_ indexed (cm^2^/m^2^)	4.54 (1.11)	4.14 (1.19)	0.072
Area AA_min_ indexed (cm^2^/m^2^)	4.02 (1.09)	3.44 (1.16)	0.008
Aortic arch length (mm)	125 (20.9)	118 (18.2)	0.068
**Aortic stiffness**			
PWV (m/s)	6.25 (1.54)	5.24 (1.57)	0.003
AA distensibility (10^−3^ kPa^−1^)	2.72 (1.1)	5.50 (1.5)	<0.001
**Aortic flow parameters**			
Global aortic net volume (mL)	59.69 (17.0)	62.04 (19.1)	0.494
Regurgitation volume V_Reg_ (mL)	13.17 (8.05)	0.71 (0.57)	<0.001
Regurgitation fraction (%)	13.75 (8.39)	0.33 (0.08)	<0.001
Backward peak flow rate Q_BFpeak_ (mL/s)	51.47 (21.8)	43.87 (20.2)	<0.001
Backward volume V_BF_ (mL)	16.70 (11.4)	9.59 (4.4)	0.001
Backward flow appearance time t_BF_ (ms)	147 (73.7)	166 (79.8)	0.052
Backward peak flow rate time (ms)	283 (75.6)	303 (59.4)	0.057
Forward peak flow rate Q_FFmax_ (mL/s)	238.42 (73.9)	297.71 (85.4)	<0.001
Forward volume V_FF_ (mL)	75.84 (20.1)	70.87 (22.3)	0.027
Q_BFmax_/Q_FFmax_ ratio (%)	21.40 (9.2)	14.49 (6.3)	<0.001
V_BF_/V_FF_ ratio (%)	22.02 (18.7)	13.60 (8.2)	<0.001

Abbreviations: AA, ascending aorta; BAV, bicuspid aortic valve; Q_BFmax_, peak backward flow rate; AA-Vreg, ascending aortic regurgitation volume; AA-V_BF_, ascending aortic backward flow volume; tBF, backward flow appearance time; Q_FFmax_, peak forward flow rate; V_FF_, forward flow volume; PWV, pulse wave velocity.

**Table 3:** Multivariable linear regression models evaluating variables associated with the peak backward-to-forward flow-rate ratio.

	Q_BFmax_/Q_FFmax_		
	β ± SE	Individual (*p*)	Overall R^2^ (*p*)
**Model A**			
Age	−0.214 ± 0.114	0.0621	0.21 (<0.001)
Sex	−4.091 ± 1.013	0.0001	
Body surface area	−2.196 ± 2.586	0.3972	
**Model B**			
Age	−0.268 ± 0.117	0.0245	0.43 (<0.001)
Sex	−4.096 ± 1.018	0.0001	
Body surface area	−1.926 ± 2.637	0.4717	
AA_max_ area	0.656 ± 0.891	0.4669	
AA_min_ area	−0.677 ± 0.887	0.4471	
AA-V_Reg_	0.113 ± 0.048	0.0021	
**Model C**			
Age	−0.235 ± 0.112	0.0848	0.49 (<0.001)
Sex	−3.979 ± 0.977	0.0001	
Body surface area	−1.933 ± 2.493	0.4237	
LV-GLS	0.517 ± 0.049	0.0046	
LV-torsion	2.966 ± 1.488	0.0488	
**Model D**			
Age	−0.248 ± 0.111	0.0387	0.52 (<0.001)
Sex	−4.126 ± 0.966	0.0001	
Body surface area	−2.326 ± 2.461	0.4399	
AA-V_Reg_	0.173 ± 0.049	0.0381	
LV-GLS	0.469 ± 0.229	0.0271	

Note: Regression coefficients (β ± standard error) are given for Q_BFmax_/Q_FFmax_. Adjustment models: (1) Model A: age, sex, and body surface area; (2) Model B: Model A with the addition of AAmax area, AAmin area, and AA-V_Reg_; (3) Model C: Model A with the addition of LV-GLS and LV torsion; and (4) Model D: Model A with the addition of AA-V_Reg_ and LV-GLS. These models were exploratory and were intentionally limited in the number of covariates because of the modest BAV sample size and potential multicollinearity among related haemodynamic variables. Associations should be interpreted within the specified models. Abbreviations: AA, ascending aorta; AAmax, maximal ascending aortic area; AAmin, minimal ascending aortic area; AA-V_Reg_, ascending aortic regurgitation volume; LV-GLS, left ventricular global longitudinal strain; LV-torsion, left ventricular torsion; Q_BFmax_, peak backward flow rate; Q_FFmax_, peak forward flow rate.

## Data Availability

The data supporting the findings of this study are available from the corresponding author upon reasonable request. The data are not publicly available due to privacy and ethical restrictions related to participant information.
